# Sex-specific differences in the experience of adverse childhood experiences: transmission, protective, and risk factors from the perspectives of parents and their children-results of an 18-year German longitudinal study

**DOI:** 10.1186/s13034-025-00904-6

**Published:** 2025-04-26

**Authors:** Max Supke, Kurt Hahlweg, Wolfgang Schulz, Ann-Katrin Job

**Affiliations:** 1 Institute of Psychology, Department of Clinical Psychology, Psychotherapy and Diagnostics, Technische Universität Braunschweig, Humboldtstr. 33, 38106 Braunschweig, Germany; 2 Leibniz Institute for Resilience Research (LIR), Mainz, Germany; 3 Institute of Psychology, Universität Kassel, Kassel, Germany

**Keywords:** Adverse childhood experiences (ACE), Prevention, Longitudinal, Sex-specific differences, Intergenerational transmission, Risk factors

## Abstract

**Theoretical background:**

Adverse childhood experiences (ACEs) are strongly associated with mental and physical health problems across the lifespan, emphasizing the critical need for prevention. Sex-specific differences in both the prevalence and long-term consequences of ACEs have rarely been analyzed, especially in longitudinal studies, which are particularly needed.

**Objective:**

This longitudinal study explores risk and protective factors as well as the intergenerational transmission of ACEs from parents to children, with a focus on sex-specific effects.

**Methods:**

Data from 316 families participating in the 18-year German longitudinal project “Future Family” were analysed. The dataset included information from mothers (54 years), fathers (57 years), and their emerging adults (22 years).

**Results:**

Daughters and mothers reported significantly more ACEs than fathers and sons, particularly in the categories of abuse and neglect. Experiencing four or more ACEs was associated with higher levels of psychological distress and lower life satisfaction for both parents and children. Approximately half of the emerging adults experienced a similar number of ACEs as their parents; however, the types of ACEs often differed, with children encountering distinct ACEs. Protective factors, such as higher maternal socio-economic status, maternal participation in the Positive Parenting Program (Triple P), and fewer internalizing problems in early childhood, were associated with a reduced number of ACEs in children by the age of 18.

**Conclusion:**

Although women report higher rates of ACEs, men are not less affected in terms of psychological distress. Sex-specific considerations appear to be crucial in the prevention of ACEs and should be integrated into targeted strategies. Our findings highlight the importance of considering both parents’ perspectives in developing and implementing effective preventive interventions in families.

## Introduction

 Mental health problems and disorders are globally prevalent and pose significant challenges to health systems. Most mental disorders manifest during adolescence or emerging adulthood. Psychological research shows that approximately 50% of all mental health problems begin before the age of 14, and 75% before the age of 24 [1]. Therefore, childhood, adolescence, and emerging adulthood are critical developmental periods for mental health [2]. Experiencing adverse events, such as abuse or neglect during childhood and adolescence, appears to play a critical role in the development of mental health problems across the lifespan.

The CDC-Kaiser Permanente Adverse Childhood Experiences (ACE) Study [3], conducted by Felitti et al. in 1998 [4], was a pioneering investigation into ACEs in the United States. This study surveyed almost 17,000 Americans, examining 10 types of ACEs, including sexual abuse, neglect, domestic violence, and divorce. The findings revealed the widespread occurrence of ACEs and their long-term associations with various mental and physical health outcomes. Numerous subsequent studies have confirmed that ACEs are prevalent worldwide, not just in the U.S. (e.g., [5]).

## Adverse childhood experiences: prevalence and subsequent outcomes

Extensive meta-analyses and reviews consistently confirm the negative long-term effects of ACEs throughout the lifespan (e.g., [6–9]). ACEs are not rare phenomena; children and adolescents around the world frequently encounter them. Based on their meta-analysis, Hillis, Mercy, Amobi, and Kress [10] estimated that over one billion children aged 2 to 17 were victims of violence worldwide in 2015. Hughes et al. [6] conducted a meta-analysis addressing the global prevalence and comorbidity of ACEs, involving 253,719 participants from 37 studies. Of these participants, 57% retrospectively reported experiencing at least one ACE, while 13% reported four or more ACEs by the age of 18. Experiencing ≥ 4 ACEs was considered a significant risk factor because it showed substantial associations with 23 outcome variables related to physical and mental health. These outcomes included physical illnesses such as diabetes (OR = 1.5) and cancer (OR = 2.3), as well as mental health problems including anxiety (OR = 3.7), depression (OR = 4.4), problematic drug use (OR = 10.2), and suicide attempts (OR = 30.1). Studies from various countries, such as England [11], Canada [12], different Balkan countries [13], and China [14], also suggest that ACEs are globally prevalent.

Since the study at hand focuses on data from Germany, the prevalence rates of ACEs within the German population are discussed. Witt et al. [15] found that 44% of respondents in a representative German sample (*N* = 2,531; age range: 14 years and older; mean age *M* = 49 years, *SD* = 18) reported having experienced at least one ACE. The study utilized the ACE-D, a German version of the Adverse Childhood Experiences Questionnaire [4, 16], to assess 10 different ACEs in adults up to the age of 18. The most frequently reported ACEs included divorce/parental separation (19%), parental alcohol/drug abuse (17%), emotional neglect (13%), and emotional abuse (13%). 9% of participants reported to have experienced ≥ 4 ACEs. This at-risk group demonstrated higher rates of depressive symptoms (OR = 7.8), increased anxiety symptoms (OR = 7.1), and reduced life satisfaction (OR = 5.1). These findings highlight that the prevalence of ACEs is significant in Germany as well.

## Sex-specific differences in adverse childhood experiences

In addition to the general occurrence of ACEs, it is crucial to consider sex-specific differences. Women are more likely to report higher rates of ACEs than men (e.g., [9]). According to the U.S. Child Maltreatment Report, the victimization rate for girls was higher, at 8.7 per 1,000 individuals, compared to 7.5 per 1,000 individuals for boys in 2021 [17]. Another U.S. study by Haahr-Pedersen et al. [18] found significant sex differences, with females more likely to report experiences such as sexual abuse, physical neglect, emotional neglect, alcohol and drug abuse in the household, and having a household member with a serious mental illness. The authors also demonstrated that females and males exhibit distinct patterns of ACEs, with females potentially experiencing more complex and varied patterns.

In Germany, research on sex-specific differences in ACEs is rare (e.g., [19–21]). A study by Häuser et al. [19] found that female sex is a predictor for experiencing sexual abuse and for severe forms of sexual abuse. Another study by Witt et al. [21] found that females in the general population seem to be at a greater risk for sexual and emotional abuse compared to males.

Considering sex-specific aspects is crucial because studies indicate sex-specific differences in the analysis of associations between ACEs and health outcomes or risk behaviors, such as smoking or alcohol use. For instance, a U.S.-study by Strine et al. [22] found that psychological distress mediated the relationship between ACEs and adult smoking among women. This association was not significant among men. Thompson et al. [23] observed that childhood exposure to parental separation, household drug abuse, household problem drinking, or physical abuse was associated with smoking in both men and women. Sexual and verbal abuse, however, showed significant associations with smoking in females but not in males. Another study by Leban and Gibson [24] initially found no sex-specific differences in the experience of ACEs. Delinquency, however, could be significantly predicted by ACEs for boys (assessed two and a half years earlier), while substance use (alcohol, nicotine, marijuana) could only be predicted by ACEs for girls. These findings highlight the importance of distinguishing between sexes in ACE research.

## Familial transmission of adverse childhood experiences

Hughes et al. [6] identified a potential cyclic pattern of ACEs within the family context. Specifically, children who have experienced multiple ACEs are more likely to engage in problematic alcohol or drug use during adolescence and young adulthood [22, 23, 25] to cope with the emotional aftermath of their ACEs (e.g., to reduce intense depressive and/or anxiety symptoms through substance use; [4, 6]). This, in turn, increases the risk of resorting to violence, as substance use diminishes inhibitions. The formation of a new family may then perpetuate the cycle, with the alcohol- or drug-dependent parent (formerly the affected child) being more prone to engage in violent behaviours, thereby causing the recurrence of ACEs in the new family (e.g., [26]). These intergenerational connections set the stage for a potentially enduring cycle within affected families, spanning multiple generations.

A meta-analysis by Madigan et al. [27] demonstrated that intergenerational transmission of child maltreatment exists, although with a modest effect size (*d* = 0.45). This suggests that many families successfully break the cycle of maltreatment. Factors such as safe, stable, and nurturing relationships with parents, siblings, and partners can play a crucial role in this process [28]. Several studies have established an association between the number of ACEs experienced by mothers and the mental health of their children [29–31]. However, there is a notable lack of studies examining whether the same ACEs persist across multiple generations within a family. To address this gap, a study by Bunting et al. [32] explored the continuity of adversity across generations using data from the Northern Ireland Youth Wellbeing Survey, encompassing 1,042 pairs of parents and adolescents aged 11–19 years. The correlation between the ACE scores of children and their parents, while statistically significant, was relatively weak (*r* =.13, *p* <.01). The authors reported a rate of intergenerational continuity/discontinuity of any ACEs of approximately 56%/44%. In their hierarchical regression model, factors such as receiving household benefits, poor child health, perceiving lower family support, fewer children in the household, older child age, and younger parent age were significantly associated with child ACE exposure.

The current research landscape on ACEs in Germany reflects a notable lack of studies, particularly of longitudinal studies, examining the consistency of ACE reports between parents and their children [9, 33].

## The prevention of adverse childhood experiences

According to the U.S. Centers for Disease Control and Prevention, ACEs are considered preventable. In the United States, various frameworks have been developed to address and prevent ACEs (e.g., [34, 35]). These frameworks emphasize the critical role of strengthening family relationships and recommend intervention and prevention programs. An example is the comprehensive technical package proposed by Fortson et al. [35], which encompasses a range of policy, normative, and programmatic activities aimed at preventing ACEs and fostering healthier family environments. The primary strategies outlined in this package include: strengthening economic supports to families (e.g., implementing family-friendly work policies), fostering a change in social norms to support parents and positive parenting (e.g., through public engagement and education campaigns), ensuring quality care and education early in life (e.g., improving child care quality through licensing and accreditation), enhancing parenting skills to promote healthy child development (e.g., through parenting skill and family relationship approaches), and intervening to mitigate harm and prevent future risks (e.g., implementing behavioral parent training programs). The reported evidence suggests that parenting training programs can be helpful in preventing ACEs.

The 45-year longitudinal Dunedin Study has been particularly insightful in advancing the development of preventive measures, offering valuable insights into the long-term effects of ACEs on later health. The study revealed that the associations between ACEs in childhood and health outcomes three decades later were mediated by several factors, notably, more stressful life events, more perceived stress, more negative emotionality, and engagement in unhealthier behaviours (such as smoking, unhealthy diet, no or few physical activity, and alcohol consumption). Based on these findings, the authors suggest that public health efforts could effectively focus on addressing these four aspects to mitigate the later health consequences of ACEs, since ACEs are no longer preventable once they have occurred [36].

For the prevention of ACEs across generations, it is important to understand intergenerational processes in the familial transmission of ACEs, although data from parents and children at the same time are rarely available [33].

## The present study

Given the importance of family factors in the prevention of ACEs, this 18-year German longitudinal study examines ACEs among parents and their young adult children. Due to the lack of ACE-focused studies in Germany, formulating precise hypotheses is not possible. Therefore, this study addresses the following research questions:

1. Are there sex-specific differences between (a) daughters and sons as well as (b) mothers and fathers concerning the prevalence of ACEs in this German study sample?
2. What is the stability of ACEs from one generation to the next (intergenerational transmission)? Do the children experience the same ACEs as their parents (continuity)?
3. Are there sex-specific differences in the associations between experiencing ACEs and health outcomes in (emerging) adulthood?
4. Are there any familial factors that can prevent the occurrence of ACEs in the next generation of children? Based on the current state of research, we examined socioeconomic status, participation in a parenting program, parental and child mental health problems, and dysfunctional parenting as potential risk and protective factors.

## Methods

### Sample recruitment

The “Future Family” (FF) project, initiated in 2001/2002, comprises two sub-studies. The FF I-study [37] was conducted as a randomized controlled trial, while the FF II-study [38] was a non-controlled open trial. Both studies aimed to evaluate the effectiveness of the Positive Parenting Program (Triple P; [39]). The motivation behind conducting the FF II-study was the underrepresentation of families from lower socioeconomic statuses in the FF I-study. The FF III-study represents a 10-year follow-up (FU10; [40]), while the FF IV-study constitutes an 18-year follow-up (FU18) of the FF I and FF II-studies. These follow-up studies used a longitudinal design to examine the long-term effectiveness of the Triple P and to predict mental health outcomes among participants as they transitioned into adolescence and emerging adulthood. The analyses considered various risk and protective factors identified during their kindergarten years.

For the initial assessment (Pre), families with children aged 2.5 to 6 years were recruited from 17 kindergartens in Braunschweig (a large German city). The original sample comprised *N* = 477 families (FF-I: *n* = 280 families, FF-II: *n* = 197 families). At Pre, the mothers’ average age was *M* = 35.2 years (*SD* = 5.0), the fathers’ average age was *M* = 38.8 years (*SD* = 6.0), and the children’s average age was *M* = 4.1 years (*SD* = 1.0). A total of *n* = 458 families participated in the one-year follow-up (FU1; retention rate: 96%); *n* = 449 families participated in the two-year follow-up (FU2; 94.1%); *n* = 361 families continued to participate after 10 years (FU10; 75.7%), and *n* = 316 families participated in the 18-year follow-up (FU18; retention rate: 67.1%, six of the original 477 families were not included as they did not meet the inclusion criteria).

### Sample characteristics (FU18)

At FU18 (*n* = 316), the mean age of emerging adults was 22.3 years (*SD* = 1.2), the mean age of mothers was 53.5 years (*SD* = 4.8), and the mean age of fathers was 56.5 years (*SD* = 4.9). School education of mothers/fathers: without a school leaving certificate/9 classes 9%/15%, 10 classes 37%/24%, A-levels/high school 55%/61%. Only 2% of the families had a low socioeconomic status, 46% had a middle socioeconomic status, and 52% had a high socioeconomic status. About one in five families (19%) had a migration background. The sex ratio was almost equal for the emerging adults (48% girls/52% boys). School education of emerging adults: without a school leaving certificate/9 classes 10%, 10 classes 18%, and A-levels/high school 73%. Two thirds (67%) of the families in this sample took part in the Triple P as part of the experimental condition. For this reason, the data are from an intervention study sample.

There were notable differences in sociodemographic data at the pre-assessment between families who participated at FU18 and those who did not (dropouts). The dropouts were more likely to be single parents (*p* <.001), more frequently had a low socio-economic background (indicated by the school leaving qualifications of mothers and fathers, Social Structure Index of their child’s kindergarten, and monthly household income; each *p* <.001), and were more often characterized by a migration background (*p* =.036). Additionally, drop-out mothers were significantly younger compared to those mothers who participated at FU18 (*p* <.001) and reported significantly more symptoms of psychopathology at baseline (*p* =.027). As a result, the representativeness of the FU18 sample is limited when compared to the total baseline sample.

## Procedure

The FU18 survey involved a combination of structured personal interviews (e.g., to assess sociodemographic data) conducted separately with parents and emerging adults, along with a set of standardized questionnaires. Up until FU10, these interviews took place in person during home visits. However, due to the COVID-19 pandemic, FU18 assessments were predominantly conducted via telephone and online through the SurveyMonkey survey platform (https://www.surveymonkey.de). To compensate for their time and participation in the approximately 2.5-hour survey, both emerging adults and parents received 50€ each. Prior to the survey, written informed consent was obtained from all participants, and the study adhered to the principles outlined in the Declaration of Helsinki. Ethical approval was granted by the ethics committee of the German Psychological Society (DGPs; identification number: WS 12_2010) and the independent ethics committee of Technische Universität Braunschweig (identification number D-2019-01; Faculty of Life Sciences).

## Measures

*Adverse childhood experiences (ACE-D; FU18)* The ACE-D [16] serves as the German adaptation of the Adverse Childhood Experiences Questionnaire [4]. Participants answered the ten items dichotomously, with 1 = *yes* or 0 = *no*. Individual scores are then totalled, resulting in a cumulative score ranging from 0 to 10. This score reflects the number of potentially traumatic events experienced during childhood and adolescence up to the age of 18. At FU18, the ACE-D was filled out by mothers, fathers, and young adults. In the present sample, the internal consistency was α = 0.76 for mothers, α = 0.73 for fathers, and α = 0.73 for young adults.

*Dysfunctional parenting practices (EFB; Pre)* The German version [41] of the Parenting Scale [42] was used to assess dysfunctional parenting. Parents rated the 35 items on a 7-point Likert scale, reflecting their engagement in dysfunctional parenting practices. Example items include, “When my child does something I don’t like… (1)…I do something about it every time” or “ (7)…I just let it pass.” The individual item scores were averaged to derive a mean total score, with higher scores indicating a greater use of dysfunctional parenting practices, such as overreaction. The internal consistency of the total score was deemed satisfactory (Pre: α mothers = 0.87, α fathers = 0.86).

*Parental symptoms of depression*,* anxiety*,* and stress (DASS; Pre)* Symptoms of depression, anxiety, and stress in mothers and fathers were assessed using the German version of the Depression Anxiety Stress Scales ([43]; German version: [44]). This self-report questionnaire comprises 42 items that assess symptoms related to the emotional states of depression, anxiety, and stress, with 14 items allocated to each category (e.g., I felt I was close to panic.– 0 = *Did not apply to me at all* or 3 = *Applied to me very much or most of the time*). A higher total score indicates a greater experience of symptoms in the past four weeks. The internal consistency in our sample was rated as excellent (Pre: α mothers = 0.95/α fathers = 0.93).

*Child mental health problems in kindergarten (CBCL; Pre)* The FF-project adopted the concept of evidence-based multimodal assessment [45]. The Achenbach System of Empirically Based Assessment (ASEBA; [46]) was chosen for its suitability in longitudinal research. This system allows the assessment of mental health problems in a developmentally appropriate manner through parent reports using the Child Behavior Checklist (CBCL). The German versions of the CBCL were employed to assess mental health problems in kindergarten at Pre from the perspectives of mothers and fathers. Parents were presented with 110 items (e.g., “Argues or disagrees a lot”) and asked to rate the frequency of certain behaviors exhibited by their child on a 3-point Likert scale (0 = *not true*; 1 = *somewhat or sometimes true*; 2 = *very true or often true*). Scores for internalizing (e.g., symptoms of anxiety) and externalizing mental health problems (e.g., aggressive behavior) were formed based on the CBCL, where higher scores indicate more mental health problems in the respective area. The internal consistencies were deemed satisfactory (α mothers = 0.94, α fathers = 0.96).

### Statistical analyses

Data on the ACE-D were available for 290 young adults, 268 mothers, and 137 fathers from the 316 participating families at FU18. These data were included in the following statistical analyses. As a first step, the prevalence rates of ACEs from this study were compared with those from the representative German survey by Witt et al. [15] to determine if our sample is also representative for the general German adult population. Sex-specific differences in the number of ACEs, which were not reported in the previous study, were calculated using *χ²*- and *t*-tests (Research Question 1). In the next step, sex-specific intergenerational transmission rates of ACEs were examined, focusing on whether children experienced the same ACEs as their parents (Research Question 2). To address research question 2, the following descriptive approach was employed: The initial sample comprised mothers and fathers who had experienced specific ACEs, representing 100% of the sample. The analysis then focused on the frequency with which their children also reported the same types of ACEs. To describe the transgenerational transmission rate (continuity), the following categorization was developed for this study: 0.0% = no transmission, 0.1 − 33.2% = low transmission, 33.3 − 66.6% = moderate transmission, 66.7 − 100.0% = high transmission. Again, in the next step, sex-specific differences in varying psychological mental health outcome variables (anxiety symptoms, depressive symptoms, and life satisfaction) in adulthood were analyzed using descriptive analyses and *t*-tests (Research Question 3). To identify early childhood risk and protective factors associated with the occurrence of ACEs (Research Question 4), in the last step, multiple linear regression models were performed. The number of self-reported ACEs by young adults (up to age 18) served as the dependent variable. The assumption of multicollinearity was not violated, as all variance inflation factor (VIF) values were below 10.

## Results

### Comparison of the prevalences of this sample with a German representative sample

A comparison of the frequencies of ACEs in Table 1 shows that mothers in the current sample reported higher prevalence rates of almost all ACEs compared to the Witt et al. [15] sample. The values reported by fathers are roughly consistent with the expected rates. For children, most values align with the German comparative study; however, the prevalence of the ACE “mental illness in the household” in our sample is more than twice as high than as in Witt et al. (10.6% vs. 23.1%). The representativeness of the prevalence of ACEs in our sample therefore appears to be particularly limited in mothers.

**Table 1:** Comparison of the prevalence rates of this sample with the German representative sample from Witt et al. [15]

	Emotional abuse	Physical abuse	Sexual abuse	Emotional neglect	Physical neglect	Parental divorce/separation	Witnessed domestic violence	Alcohol and drug abuse in the household	Mental illness in the household	Incarcerated family member
Witt et al. (2019)	12.5	9.1	4.3	13.4	4.3	19.4	9.8	16.7	10.6	3.5
Mothers	25.0	17.2	14.2	26.1	6.0	25.4	5.2	23.1	19.0	2.6
Fathers	12.4	9.5	0.7	8.8	1.5	21.2	5.1	10.9	8.8	2.9
Children	14.5	5.5	5.9	19.3	4.8	23.4	3.8	14.1	23.1	2.8

The values are reported in percentages (%)

### Research question 1: sex-specific differences in the number of experienced adverse childhood experiences

When comparing daughters and sons, we found that daughters (*M* = 1.5, *SD* = 2.1) reported significantly more ACEs on average than sons (*M* = 0.8, *SD* = 1.1), with a small effect size (*t *(288) = 3.5, *p* <.001, *d* = 0.41). Detailed statistics for the ten individual ACEs assessed with the ACE-D are presented in Table 2. The table indicates that daughters were significantly more likely to report emotional abuse (Daughters: 18.6% vs. Sons: 10.3%; *p* =.045), sexual abuse (D: 10.3% vs. S: 1.4%; *p* =.001), emotional neglect (D: 28.3% vs. S: 10.3%; *p* <.001), and physical neglect (D: 7.6% vs. S: 2.1%; *p* =.028). Additionally, a significantly higher proportion (χ² (1, 290) = 12.3, *p* =.002, Phi = 0.21) of daughters (15.2%) were in the at-risk group (≥ 4 ACEs) compared to sons (3.4%).

There were even more differences when comparing mothers and fathers. On average, mothers (*M* = 1.6, *SD* = 2.0) reported significantly more ACEs than fathers (*M* = 0.8, *SD* = 1.5), (460) = 4.7, *p *<0.001, *p* = 0.44). Mothers were significantly more likely to report emotional abuse (Mothers: 25.0% vs. Fathers: 12.4%, *p* = 0.003), physical abuse (M: 17.2% vs. F: 9.5%, *p* = 0.037), and sexual abuse (M: 14.2% vs. F: 0.7%, *p* < 0.001), emotional neglect (M: 26.1% vs. F: 8.8%, *p* < 0.001), physical neglect (M: 6.0% vs. F: 1.5%, *p* = 0.037), substance use (M: 23.1% vs. F: 10.9%, *p* = 0.003) and mental illness in the household (M: 19.0% vs. F:8.8%, *p* = 0.007). Furthermore, mothers (14.9%) were significantly more likely to be in the at-risk group (≥ 4 ACEs) compared to fathers (8.0%) (χ² (1, 405) = 24.3, *p* <.001, Phi = 0.25).

**Table 2:** Frequencies and significance tests of individual aces in daughters (*n* = 145), sons (*n* = 145), mothers (*n* = 268), and fathers (*n* = 137)

ACE	Daughters in %	Sons in %	Statistics	Mothers in %	Fathers in %	Statistics
Emotional abuse	**18.6**	**10.3**	**χ² (1**,** 290) = 4.0** ***p*** **=.045** **Phi = − 0.12**	**25.0**	**12.4**	**χ² (1**,** 405) = 8.7** ***p*** **=.003** **Phi = − 0.15**
Physical abuse	7.6	3.4	χ² (1, 290) = 2.4 *p* =.123 Phi = − 0.09	**17.2**	**9.5**	**χ² (1**,** 405) = 4.4** ***p*** **=.037** **Phi = − 0.10**
Sexual abuse	**10.3**	**1.4**	**χ² (1**,** 290) = 10.6** ***p*** **=.001** **Phi = − 0.19**	**14.2**	**0.7**	**χ² (1**,** 405) = 18.8** ***p*** **<.001** **Phi = − 0.22**
Emotional neglect	**28.3**	**10.3**	**χ² (1**,** 290) = 15.0** ***p*** **<.001** **Phi = − 0.23**	**26.1**	**8.8**	**χ² (1**,** 405) = 16.9** ***p*** **<.001** **Phi = − 0.20**
Physical neglect	**7.6**	**2.1**	**χ² (1**,** 290) = 4.8** ***p*** **=.028** **Phi = − 0.13**	**6.0**	**1.5**	**χ² (1**,** 405) = 4.3** ***p*** **=.037** **Phi = − 0.10**
Parental divorce/separation	26.2	20.7	χ² (1, 290) = 1.2 *p* =.268 Phi = − 0.07	25.4	21.2	χ² (1, 405) = 0.9 *p* =.348 Phi = − 0.05
Witnessed domestic violence	5.5	2.1	χ² (1, 290) = 2.3 *p* =.127 Phi = − 0.09	5.2	5.1	χ² (1, 405) = 0.0 *p* =.961 Phi = − 0.00
Alcohol and drug abuse in the household	16.6	11.7	χ² (1, 290) = 1.4 *p* =.238 Phi = − 0.07	**23.1**	**10.9**	**χ² (1**,** 405) = 8.7** ***p*** **=.003** **Phi = − 0.15**
Mental illness in the household	26.9	19.3	χ² (1, 290) = 2.4 *p* =.125 Phi = − 0.09	**19.0**	**8.8**	**χ² (1**,** 405) = 7.3** ***p*** **=.007** **Phi = − 0.13**
Incarcerated family member	3.4	2.1	χ² (1, 290) = 0.5 *p* =.473 Phi = − 0.04	2.6	2.9	χ² (1, 405) = 0.3 *p* =.857 Phi = 0.01
0 ACE	**44.8**	**46.9**	**χ² (1**,** 290) = 12.3** ***p*** **=.002** **Phi = 0.21**	**38.4**	**64.2**	**χ² (1**,** 405) = 24.3** ***p*** **<.001** **Phi = 0.25**
1–3 ACEs	**40.0**	**49.7**		**46.6**	**27.7**	
≥ 4 ACEs	**15.2**	**3.4**		**14.9**	**8.0**	
Number of experienced ACEs (*M* and *SD*)	**1.5**	**0.8**	***t*** **(288) = 3.5** ***p*** **<.001** ***d*** **= 0.41**	**1.6**	**0.8**	***t*** **(403) = 4.7** ***p*** **<.001** ***d*** **= 0.44**

Significant results are shown in bold

### Research question 2: do children experience the same adverse childhood experiences as their parents?–intergenerational transmission and continuity

To clarify the descriptive statistical approach (used in Table 3), consider the following example: Among the mothers in our sample, 33 reported having experienced emotional abuse. Of these mothers’ daughters, 8 also reported to have experienced emotional abuse. This corresponds to a transmission rate of 8/33 = 24.2%, which falls into the “low transmission” category.

**Table 3:** Intergenerational transmission rates of experienced aces between parents and their children

	Mothers *n*	→	Daughters *n* (trans. rate)	Mothers *n*	→	Sons *n* (trans. rate)	Fathers *N*	→	Daughters *n* (trans. rate)	Fathers *n*	→	Sons *n* (trans. rate)
Emotional abuse	33	→	8 (24.2%) Low	28	→	2 (7.1%) Low	11	→	2 (18.2%) Low	6	→	1 (16.7) Low
Physical abuse	23	→	3 (13.0%) Low	18	→	1 (5.6%) Low	8	→	0 (0.0%) None	5	→	0 (0.0%) None
Sexual abuse	19	→	3 (15.8%) Low	16	→	1 (6.3%) Low	1	→	0 (0.0%) None	0	→	NA
Emotional neglect	33	→	16 (48.5%) Moderate	30	→	1 (3.3%) Low	8	→	2 (25.0%) Low	2	→	0 (0.0%) None
Physical neglect	10	→	3 (30.0%) Low	4	→	0 (0.0%) None	0	→	NA	2	→	0 (0.0%) None
Parental divorce/separation	27	→	9 (33.3%) Moderate	35	→	12 (34.3%) Moderate	18	→	3 (16.7%) Low	10	→	2 (20.0%) Low
Witnessed domestic violence	9	→	0 (0.0%) None	4	→	0 (0.0%) None	6	→	0 (0.0%) None	1	→	0 (0.0%) None
Alcohol and drug abuse in the household	33	→	9 (27.3%) Low	22	→	2 (9.1%) Low	6	→	3 (50.0%) Moderate	9	→	3 (33.3%) Moderate
Mental illness in the household	28	→	11 (39.3%) Moderate	17	→	6 (35.3%) Moderate	5	→	2 (40.0%) Moderate	7	→	5 (71.4%) High
Incarcerated family member	3	→	2 (66.7%) High	2	→	0 (0.0%) None	0	→	NA	4	→	1 (25.0%) Low
0 ACE	56	→	33 (58.9%) Moderate	40	→	23 (57.5%) Moderate	43	→	22 (51.2%) Moderate	39	→	24 (61.5%) Moderate
1–3 ACEs	43	→	14 (32.6%) Low	72	→	39 (54.2%) Moderate	20	→	10 (50.0%) Moderate	15	→	7 (46.7%) Moderate
≥ 4 ACEs	25	→	8 (32.0%) Low	11	→	0 (0.0%) None	6	→	1 (16.7%) Low	5	→	0 (0.0%) None

*NA* Not applicable, *trans. rate* transgenerational transmission rate (continuity); the following categorization was used: 0.0% = none/no transmission, 0.1 − 33.2% = low transmission, 33.3 − 66.6% = moderate transmission, 66.7 − 100.0% = high transmission

Overall, transmission rates between parents and children were rather low for most ACEs. Out of the 40 available transmission rates between parents and children: 16 (40.0%) were in the *low* range, 8 (20.0%) were in the *moderate* range, and only 2 (5.0%) were in the *high* range. For 11 ACEs (27.5%), *no transmission* at all was observed and for 3 (7.5%) ACEs related to fathers, there were no fathers reporting these specific ACEs (NA).

The highest level of transmission rates was observed between mothers and daughters (None: 10.0%, Low: 50.0%, Moderate: 30.0%, and High: 10.0%). There were *moderate* transmission rates for experienced emotional neglect (48.5%), divorce/separation of parents (33.3%), and mental illness in the household (39.3%), while *high* transmission rates were found for the ACE “incarcerated family member” (66.7%). Between mothers and sons (None: 30.0%, Low: 50.0%, Moderate: 20.0%, and High: 0.0%), no high transmission rates were found while *moderate* rates were found for experienced divorce/separation of parents (34.3%) and mental illness in the household (35.3%).

The overall transmission rates from fathers to both daughters (None: 30.0%, Low: 30.0%, Moderate: 20.0%, and High: 0.0%) and sons (None: 40.0%, Low: 30.0%, Moderate: 100.0% and High: 10.0%) were lower compared to the transmission rates from mothers. *Moderate* transmission rates were found between fathers and daughters (50.0%) and sons (33.3%) regarding experienced alcohol and drug abuse in the household. Additionally, *moderate* transmission rates were found between fathers and daughters for mental illness in the household (39.3%), while *high* rates were found among sons (71.4%). Mental illness in the household thus showed moderate to high associations in all four constellations.

In terms of ACE groups, both mothers and fathers demonstrated that 0 ACEs and 1–3 ACEs generally had moderate transmission rates (32.6–61.5%). For 4 or more ACEs, the transmission rate was in the low range (0.0–30.0%).

### Research question 3: sex-specific differences in psychological outcome variables in adulthood

Among emerging adults (mean age: 22 years), daughters reported significantly more anxiety symptoms compared to sons (GAD-7- Daughters: *M* = 5.8, *SD* = 4.0 vs. Sons: *M* = 4.6, *SD* = 4.3; *t* (290) = 2.4, *p* =.017, *d* = 0.28). However, no sex-specific differences were found for depressive symptoms (PHQ-9-D: *M* = 7.0, *SD* = 5.1 vs. S: *M* = 6.2, *SD* = 5.2; *t* (290) = 1.4, *p* =.158, *d* = 0.17) and life satisfaction (FLZ-D: *M* = 54.3, *SD* = 35.1 vs. S: *M* = 50.7, *SD* = 36.5; *t* (290) = 0.9, *p* =.397, *d* = 0.10).

In the next step, outcome variables were evaluated sex-specifically within the different ACE groups. Due to small cell sizes, results are reported descriptively. Table 4 illustrates that both daughters and sons reported worsening psychological outcomes as the number of ACEs increased. Particularly, the group with ≥ 4 ACEs exhibited the worst outcomes, with symptom levels two to three times higher compared to those with no ACEs.

Among parents (mean age: 54–57 years), mothers reported significantly higher levels of depressive symptoms (PHQ-9: *M* = 5.3, *SD* = 4.4) compared to fathers (*M* = 3.5, *SD* = 3.1; *t* (442) = 4.9, *p* <.001, *d* = 0.45). Similarly, mothers reported significantly more anxiety symptoms (GAD-7: *M* = 4.2, *SD* = 3.5) than fathers (*M* = 2.7, *SD* = 2.9; *t* (442) = 4.5, *p* <.001, *d* = 0.45). No difference was however found between mothers’ and fathers’ general life satisfaction (FLZ: *M* = 62.0, *SD* = 34.1 vs. F: *M* = 66.6, *SD* = 30.0; *t* (442) = − 1.4, *p* =.161, *d* = − 0.14).

When examining the ACE groups (Table 4), scores for both depressive and anxiety symptoms again worsened as the number of ACEs increased. Mothers consistently had slightly worse outcomes compared to fathers. The group with ≥ 4 ACEs had the highest distress in both parents, with mothers reporting considerably more depressive symptoms (*M* = 9.0, *SD* = 6.1) compared to fathers (*M* = 5.0, *SD* = 4.9).

**Table 4:** Psychological outcomes of daughters and sons as well as mothers and fathers differentiated by the number of experienced aces

	Daughters			Sons		
	PHQ-9 *M* (*SD*)	GAD-7 *M* (*SD*)	FLZ *M* (*SD*)	PHQ-9 *M* (*SD*)	GAD-7 *M* (*SD*)	FLZ *M* (*SD*)
0 ACE	5.3 (4.3)	4.4 (3.6)	68.0 (28.2)	5.0 (3.9)	3.9 (3.4)	55.6 (32.5)
1–3 ACEs	7.8 (4.6)	6.4 (3.7)	46.9 (32.8)	6.9 (5.6)	4.9 (4.7)	50.2 (36.0)
≥ 4 ACEs	11.0 (6.0)	8.4 (4.7)	32.9 (43.5)	14.6 (7.3)	10.6 (5.9)	− 8.8 (48.5)
	**Mothers**			**Fathers**		
0 ACE	3.9 (2.7)	3.2 (2.4)	70.8 (31.6)	3.0 (2.6)	2.3 (2.5)	71.5 (28.3)
1–3 ACEs	5.3 (4.3)	4.4 (3.6)	60.3 (33.4)	3.8 (3.0)	2.8 (2.4)	62.8 (32.4)
≥ 4 ACEs	9.0 (6.1)	6.2 (4.8)	43.0 (36.6)	5.0 (4.9)	5.0 (4.5)	46.2 (29.1)

0 ACEs: daughters: *n* = 65, sons *n* = 68; 1–3 ACEs: daughters: *n* = 58, sons *n* = 72; ≥4 ACEs: daughters: *n* = 21, sons *n* = 5

0 ACEs: mothers: *n* = 103, fathers *n* = 86; 1–3 ACEs: mothers: *n* = 125, fathers *n* = 36; ≥4 ACEs: mothers: *n* = 40, fathers *n* = 11

### Research question 4: risk and protective factors for aces in childhood and adolescence

Maternal and paternal factors (predictors) during kindergarten age (Pre) were examined separately to predict the number of ACEs (ranging from 0 to 10 ACEs, criterion) for their daughters and sons. The results of the four models are displayed in Table 5. Only the mothers-daughters prediction model turned out to be significant (*F* [6, 128] = 4.5, *p* <.001), while the other three failed to reach significance. For daughters, a higher socio-economic status of the mother (β = − 0.21, *p* =.014), participation in the Triple P (β = − 0.19, *p* =.029), and fewer internalizing child mental health problems (β = 0.22, *p* =.044) were associated with a lower number of ACEs up to the age of 18. The maternal linear regression model explained 17% of the variance. Notably, the paternal predictive model explained 14% of the variance for sons, with externalizing behaviour problems showing a trending association with a higher number of ACEs (β = 0.31, *p* =.041).

**Table 5:** Results of the multiple linear regression models to predict the number of aces experienced by daughters and sons using maternal and paternal factors at kindergarten age (Pre)

	Models with maternal factors at Pre										Models with paternal factors at Pre									
	For daughters (*n* = 135)					For sons (*n* = 130)					For daughters (*n* = 81)					For sons (*n* = 87)				
	*B*	*SD*	β	*p*	VIF	*B*	*SD*	β	*p*	VIF	*B*	*SD*	β	*p*	VIF	*B*	*SD*	β	*p*	VIF
Constant	1.4	1.4		0.312		0.5	0.9		0.570		1.4	1.4		0.344		− 0.8	1.0		0.440	
Socioeconomic status 0 = low-middle, 1 = high	− 0.8	0.3	− 0.21	0.014	1.1	− 0.2	0.2	− 0.09	0.332	1.1	− 0.5	0.3	− 0.20	0.073	1.0	0.1	0.2	0.02	0.832	0.913
Triple P 0 = no participation, 1 = participation	− 0.8	0.4	− 0.19	0.029	1.1	0.2	0.2	0.09	0.347	1.0	− 0.5	0.3	− 0.16	0.161	1.0	0.3	0.2	0.16	0.147	1.1
Mental health problems of the parent (DASS)	0.0	0.0	0.03	0.694	1.2	0.2	0.2	0.01	0.959	1.4	0.1	0.0	0.18	0.132	1.1	0.0	0.0	0.18	0.129	1.2
Dysfunctional parenting (EFB)	0.4	0.3	0.12	0.173	1.2	0.0	0.0	0.01	0.930	1.4	− 0.1	0.3	− 0.03	0.824	1.1	0.0	0.2	− 0.01	0.926	1.2
Internalizing mental health problems of the child (CBCL– T-values)	0.4	0.1	0.22	0.044	1.8	0.0	0.0	− 0.14	0.313	2.3	0.0	0.0	0.01	0.715	1.7	− 0.0	0.0	− 0.16	0.273	2.1
Exernalizing mental health problems of the child (CBCL– T-values)	0.0	0.0	− 0.10	0.362	2.0	0.0	0.0	0.18	0.173	2.2	0.0	0.0	0.08	0.580	1.8	0.1	0.0	0.31	0.041	2.0
Model statistics	*R*² = 0.17, *F* (6, 128) = 4.5, *p* <.001					*R*² = 0.04, *F* (6, 123) = 0.8, *p =*.608					*R*² = 0.11, *F* (6, 74) = 1.6, *p =*.167					*R*² = 0.14, *F* (6, 80) = 2.2, *p =*.053				

## Discussion

The Future Family project is a longitudinal study that collected data from mothers, fathers, and children over 18 years, spanning from kindergarten to emerging adulthood. This comprehensive dataset includes information from two generations, particularly on ACEs, and therefore allowed analysis of the intergenerational transmission of these events. This is particularly important, because the findings may provide valuable insights into potential strategies in families to prevent ACEs in future generations. Since data from both mothers and fathers, as well as from daughters and sons, were available, sex-specific aspects—rarely addressed in previous research—could be analysed, while also taking into account the longitudinal timeframe for predictions spanning from kindergarten to emerging adulthood.

We initially examined whether our sample was somewhat representative of the German population regarding the occurrence of ACEs, as it was drawn from a single German city. We compared our data with the results of Witt et al. [15]. It was found that significantly more ACEs were reported by mothers than expected. One possible explanation for this discrepancy is that the mothers had been involved in our project for over 18 years. Personal interviews were typically conducted at home, which may have fostered a strong relationship with the project and lowered the threshold for disclosing sensitive past events. Another possible explanation is the lack of sex-specific comparative values for Germany, as women generally report more ACEs compared to men (e.g., [9]). The data for fathers and children were more consistent with those of the representative German sample, although the young adults significantly more often reported that one parent had suffered from a mental illness. This could be attributed to the increasing openness towards mental health issues in Germany in recent years, which has led to a decrease in stigmatization [47]. Overall, it can be concluded that the representativeness of our sample regarding the occurrence of ACEs is limited particularly among mothers.

With regard to sex-specific differences (Research Question 1), the analyses revealed that female family members, this is, both mothers and daughters, reported significantly more ACEs and experienced greater distress compared to male family members. In particular, experiences of abuse and neglect in diverse forms were reported more frequently by females. These findings are consistent with international studies suggesting that women are more likely to report these types of ACEs as well as other forms of adversity (e.g., [9, 17–19, 21]). It is furthermore noteworthy that both mothers and daughters were significantly more likely to belong to the ≥ 4 ACEs group compared to fathers and sons. Females therefore represent a high-risk population because ≥ 4 ACEs are associated with substantial psychological and physical health problems across the lifespan (e.g., [6, 15]).

Interestingly, the mean values for the frequency of ACEs and the specific frequencies for individual ACEs were relatively similar in both mothers and daughters, as well as for fathers and sons (see Table 2). Data on the familial transmission of ACEs within families as well as on factors that predict this transmission, remains limited, as such findings require longitudinal studies conducted over extended periods. To the best of our knowledge, the study by Bunting et al. [32] is the only one to report an intergenerational continuity rate of approximately 56% for ACEs for parents and adolescents. With our dataset, which includes three family members, we were able to examine the continuity of individual ACEs across two generations while also incorporating sex-specific aspects (Research Question 2). In addition, we categorized the strength of the transmission rates using a descriptive statistical approach. Analyses revealed that in our sample there was either no transmission or only low transmission rates from parents to children for most ACEs. Children therefore had a low risk of experiencing the same ACEs as their parents. When examining group membership (0, 1–3, or ≥ 4 ACEs), predominantly moderate transmission rates (31–62%) were observed, which is consistent with the findings by Bunting et al. [32]. With regard to prevention, these results suggest that although ACE group membership has a relatively high stability across two generations, there is at the same time considerable variability in the individual types of ACEs. In group of parents with ≥ 4 ACEs, however, very few and often no children also experienced ≥ 4 ACEs, which may indicate that parents who had experienced particularly high levels of adverse events might have taken extra care to prevent similar experiences for their children. Future studies should conduct more detailed analyses to identify the factors influencing which ACEs are passed down from one generation to the next.

Our findings furthermore suggest that mothers may exhibit slightly higher rates of ACE transmission to daughters compared to sons, suggesting potential sex-specific differences in transgenerational transmission as well. However, drawing similar conclusions for fathers and their children is challenging due to the small sample size of fathers in the current study. Not surprisingly, the ACE “mental illness in the household” consistently had the highest transmission rates, likely reflecting the significant hereditary and familial components of mental illness (e.g., [48, 49]). Interestingly, the transmission rate for “witnessed domestic violence” in our sample is 0%. Experiencing violence is generally considered a risk factor for perpetrating violence or engaging in dysfunctional parenting (e.g., [26]). However, greater socioeconomic resources and family support can help break the intergenerational cycle of violence [27, 28, 50]. Our analysed sample predominantly consists of individuals with a middle to high socioeconomic status, meaning these families had access to substantial resources. Additionally, families with lower socioeconomic status and those in which mothers experienced greater psychological distress were more likely to drop out of the study. This may have led to a bias in the results.

Our third research question examined sex-specific consequences of ACEs. Across all family members, an increase in the number of ACEs was associated with higher levels of depressive and anxiety symptoms as well as reduced life satisfaction. Females as well as males in the ≥ 4 ACEs group had the worst outcomes, with symptom severity two to three times higher than those with no ACEs. Sons in the ≥ 4 ACEs group reported slightly greater mental distress compared to daughters, although this may be due to the smaller sample size in this subgroup. Mothers consistently achieved slightly worse scores than fathers. Among parents, those in the ≥ 4 ACEs group were most distressed, with mothers reporting significantly higher levels of depressive symptoms than fathers. Although only a limited number of sex-specific differences were observed in our study, most findings are consistent with existing international literature, which demonstrates that ACEs are linked to poorer mental health outcomes across the lifespan (e.g., [6–9]).

Given the long-term mental health consequences of ACEs, prevention is particularly important– especially since they cannot be reversed once the adverse events occur [36]. Our sex-specific analyses reveal that a higher maternal socio-economic status, participation in the Triple P, and fewer internalizing child mental health problems (e.g., depressive and anxiety symptoms) were longitudinally associated with fewer ACEs experienced by daughters by the age of 18. These findings provide valuable starting points for prevention efforts and align with existing frameworks already implemented in the United States. As described in the introduction of this study, strengthening economic supports for families, enhancing parental skills, and fostering strong family relationships are central components of current prevention efforts (e.g., [34, 35]). The results of our prediction models provide hints that the approaches described could represent effective starting points for long-term preventive interventions. Participation in the Triple P program, in particular, appears promising, as it enhances parental competence, improves parent-child relationships, and strengthens children’s mental health within a relatively short timeframe and over a longer period of time [39]. Parent training programs not only strengthen parenting practices and the parent-child relationship but also the parental partnership [39]. Safe, stable, and nurturing relationships—whether with parents, siblings, or other family members—along with overall family support and emotional warmth, may help break the cycle of maltreatment [27, 28, 50]. A meta-analysis by Euser et al. [51] found that 5 out of 20 intervention programs were effective in preventing or reducing the risk of child maltreatment. Notably, parent training programs were associated with greater treatment effectiveness compared to interventions focusing solely on support. Moreover, from an economic perspective, such preventive interventions seem to represent a worthwhile investment, as short-term expenditures may lead to long-term economic gains [53, 54]. Our findings also suggest differential contributions of maternal and paternal factors to the variance in ACEs experienced by sons and daughters. While maternal data accounted for only 4% of the variance in sons’ ACEs (compared to 17% for daughters), paternal data explained 14% of the variance in sons and 11% in daughters, although the paternal models were not statistically significant. The lack of significance in the paternal models could be partly due to the smaller sample size of fathers. Additionally, many ACEs are linked to parenting, and in Germany, fathers were generally less involved in child-rearing at the start of the study in 2001/2002. As a result, paternal factors may not be strong long-term predictors of ACEs. However, in recent years, there has been a shift in Germany, with fathers becoming increasingly involved in parenting [52]. Therefore, further and more up-to-date studies on this aspect are necessary. These findings highlight the potential importance of incorporating data from both parents to account for sex-specific variability in ACEs and suggest that tailored approaches addressing maternal and paternal factors may be needed to optimize preventive interventions for sons and daughters.

### Strengths and limitations

The main strength of our study is the 18-year longitudinal design. As mentioned above, many children grew up with the FF project and their parents were involved for a long time, which likely lowered the threshold for both children and parents to disclose ACEs. Moreover, the longitudinal approach enables the identification of starting points for the development of preventive measures based on long-term data. Another strength is the inclusion of data from both parents, allowing for sex-specific analyses across two generations.

A major limitation of our study is the limited generalizability of the results, as our sample primarily consists of individuals from middle to upper socio-economic backgrounds, with lower socio-economic groups being underrepresented. Therefore, the findings cannot be directly applied to the general German population. In addition, our sample size is relatively small, which resulted in very small cell sizes, particularly in the paternal models, and thereby reduced the statistical power. The results are nevertheless reported as they may provide valuable insights for future studies, although the exploratory nature of the analyses should be taken into consideration. Another significant limitation is the retrospective nature of the ACEs assessment, which may have introduced recall bias, particularly among parents, as some ACEs occurred as long as 40 or more years ago. Previous studies have shown that ACEs are sometimes inaccurately remembered, potentially leading to distortions in the data [55]. Furthermore, the data (at Pre) for prediction were collected only when the children were 3–6 years old. By this time, some ACEs may have already occurred or begun. Internalizing problems became significant in the maternal model for daughters; however, increased internalizing behavioural problems in daughters could also be a consequence of earlier ACEs. Future studies should record the timing of ACEs and consider this factor in their analyses.

## Conclusion

The fact that women are more frequently affected by ACEs than men is well-documented in numerous studies, including ours. Our findings furthermore suggest that young adult men who have experienced a high number of ACEs also experience significant psychological distress—perhaps even more than young adult women. Our study also provides evidence that while the overall number of ACEs is relatively stable from one generation to the next, children often experience different ACEs than their parents. At this point, further research is needed to draw conclusions about which ACEs in parents increase the risk of specific ACEs in children. For example, a father who experienced physical neglect may have turned to alcohol at a young age, causing a parents’ substance use to become a significant ACE for the young adult. In the prevention of ACEs, sex-specific aspects appear to play a crucial role and should be taken into account. Future longitudinal studies with representative samples are needed to further explore the sex-specific aspects of ACEs, e.g., in families with a low socioeconomic background, as well as potential moderators and mediators with subsequent psychological outcomes. Additionally, studies are needed that assess predictors before the occurrence of ACEs and analyse them longitudinally.

Our findings suggest that the perspectives of both parents are important and should be considered in the development and implementation of targeted preventive strategies. Addressing the special needs of both male and female children and incorporating data from both mothers and fathers could help ensure more comprehensive and effective prevention efforts.

## Data Availability

The datasets generated and/or analyzed during the current study are not publicly available as they contain sensitive material. Furthermore, it is a longitudinal study with several assessment points. The data could possibly be used to draw conclusions about individuals. The questionnaires used can be found with the corresponding author.
